# “The Sun Do Move.”—Anti-Vaccinationists

**Published:** 1882-02

**Authors:** 


"The Sun do Move."—Anti-Vaccinationists.

Poor old John Jasper is dead. John was a sable Virginian, of devout spirit, concerned for the welfare of his brethren. John found it to be a prevailing belief that the sun was stationary. His eyes told him the contrary. He had never heard of Copernicus, but it was plain to him that a great popular delusion overspread the land. So John undertook to reform the opinions of the people, and preached sermons from the text: "The Sun do Move." And he had a large following. His arguments and demonstrations were irresistible. He had none to oppose him, for all the astronomy known to his brethren had entered their understanding through their own eyes.

The mantle of Jasper has fallen on anti-vaccinationists. Opening their tender vision on the world in which they happen to be thrown, they find society protecting itself from small-pox by vaccination. Observing a few exceptional cases of failure to protect, or of reputed evil effects, they settle the whole question mentally without looking into it further than the length of their nose. Blind with prejudice from the start, they are incapable of seeing more than one side, or one class of facts. And having once switched off the track of rational enquiry, they diverge farther and farther from it as their brains become intoxicated with the shallow draughts of muddled knowledge. After the fashion of nostrum mongers collecting testimonials, they throw their drag-net into pools and sewers, and fish up everything—fact or fiction—on the blind side of the question, carefully ignoring the incontestable and overwhelming mass of evidence on the side of vaccination. They have their societies and periodicals both in England and America, more particularly in England, where the opposition to vaccination depends, more than on anything else, on the pig-headed crosswiseness of human nature which is aroused to action by the compulsory laws. Had a law been enacted prohibiting vaccination, a majority of these crooked people would have organized on the other side and vaccinated themselves and their children at least once a year, out of sheer spite and contrariness. One honest fool in Ireland breaks up house and home to get rid of compulsory legislation, and transports his family to Pennsylvania, there to plant a colony for the infection and cultivation of small-pox among his neighbors. An English paper contains the announcement of the birth of a child who "will never be vaccinated"—the stupid father thus attempting to impose on his infant a life-long compulsion for the purpose of proclaiming his hostility to compulsion. To this crusade against common sense and universal experience, quite a number of titled individuals give the use of their names—lords and nobles, perhaps one-tenth as many as took stock in the South Sea Bubble or built up the London Perkinian Institution for the cure of disease by magnetic tractors many years ago.

Apropos to this topic, there comes to us by mail a roll of anti-vaccination literature, newspaper clippings, extracts, etc., with written references—quite a mass of interesting trash. The unknown friend who sends it has taken infinite pains with a view no doubt to the salvation of our soul. We shall preserve the curious mess for future use, with thanks for it, suggesting, however, that when written matter is wrapped and mailed as if printed, it requires letter postage, and that a conscientious advocate of reform, in anything from vaccination to polygamy, should not defraud the government.—*Pacific Medical and Surgical Journal.*

